# Colonic Foreign Body Retrieval Using a Modified TAMIS Technique with Standard Instruments and Trocars

**DOI:** 10.1155/2015/815616

**Published:** 2015-04-07

**Authors:** Shamir O. Cawich, Fawwaz Mohammed, Richard Spence, Matthew Albert, Vijay Naraynsingh

**Affiliations:** ^1^Department of Clinical Surgical Sciences, University of the West Indies, St. Augustine Campus, St. Augustine, Trinidad and Tobago; ^2^The Center for Colon and Rectal Surgery, 661 East Altamonte Drive, Altamonte Springs, FL 32701, USA

## Abstract

*Background*. Reports of retained colorectal foreign bodies (CFBs) are no longer considered uncommon. We present a case where a retained CFB was retrieved using a modified TAMIS technique using standard instruments and trocars. *Case Report*. A 52-year-old man presented with a CFB. We report our technique of extraction with standard laparoscopic instruments without specialized access platforms. *Conclusions*. This modified TAMIS technique is well suited for resource poor environments because it requires no specialized equipment, platforms, or additional skill sets compared to conventional laparoscopy.

## 1. Introduction

The earliest reports of patients presenting with retained colorectal foreign bodies (CFBs) date as far back as the 16th century [1]. Since that time, reports of retained CFBs have been increasing and it is no longer considered uncommon.

We present a case of a patient presenting with a retained CFB in which an unusual method of retrieval was used. We believe that this method is well suited for low cost environments.

## 2. Report of a Case

A 52-year-old man with no medical illnesses presented to the emergency room reporting constant pain in the gluteal region after he was allegedly abducted and beaten by a group of unknown individuals. No other useful history was volunteered.

**Figure 1:**
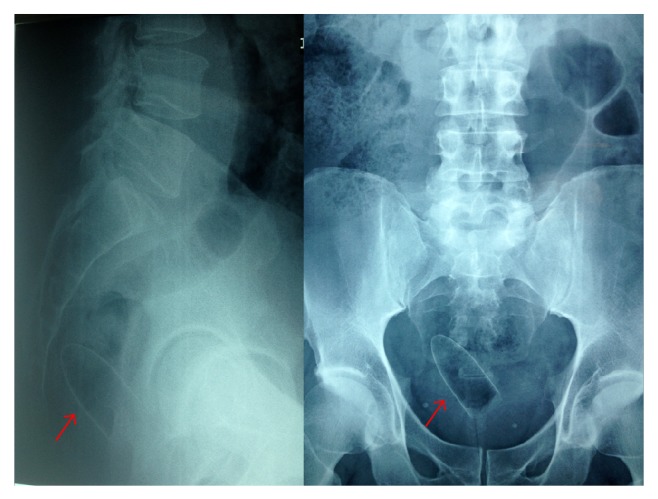
Plain radiographs of the pelvis outline a foreign body within the rectum (indicated by red arrow) that has a cylindrical shape and a tapered tip.

On examination, vital signs were normal. There were no superficial lacerations or contusions in keeping with the history of an assault. Examinations of the abdomen, chest, nervous system, and musculoskeletal system were normal. There were no external injuries on examination of the gluteal region but there was mild tenderness at the perianal region. Digital rectal examination revealed normal mucosa, an intact anal sphincter, and a normal prostate that was approximately 45 mls in volume. A plain radiograph of the pelvis was ordered as a part of the trauma series and revealed an unexpected finding: a cylindrical object with a tapered tip and radiolucent hollow base was present within the rectum (Figure 1).

With this unexpected finding, the history was revisited. Only then did the patient confess to his habit of inserting an object into the rectum for self-eroticism. The object used on this occasion was described as a metallic Cuban cigar sheath that had a tapered tip. Approximately 48 hours before, he grasped the rim of the cigar sheath with his thumb and index finger and inserted it into his rectum. However, on this occasion the cigar sheath ascended into the rectum and was not retrievable. Due to his embarrassment, he delayed presentation for 48 hours until he experienced vague rectal pain.

Although the foreign body appeared to be in the rectum on radiographs, it could not be palpated on digital rectal examination. The object was visualized on flexible sigmoidoscopy. By this time, it had ascended into the sigmoid colon but could not be retrieved since endoscopic tools could not grasp the smooth surface. An attempt to use the retroflexed scope to push the CFB distally for manual retrieval was not successful because the open end of the cigar sheath abutted the mucosa, creating a vacuum effect that kept it retained.

In order to retrieve the CFB, we secured consent for transabdominal retrieval using a laparoscopic approach. We considered retrieval by the transanal minimally invasive surgical (TAMIS) technique but we did not have specialized TAMIS platforms, instruments, or a SILS port available. Therefore, we considered inserting a laparoscope into the anus to visualize the CFB.

**Figure 2:**
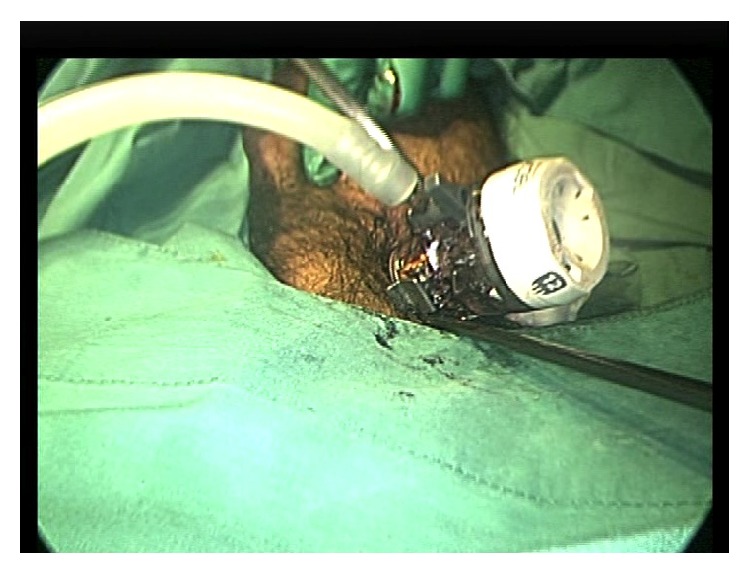
A photograph of the perineum. The patient is in lithotomy position and surgical drapes are applied. A 12 mm laparoscopic trocar is inserted into the anus and the trocar sleeve is fully advanced until the adjustable sleeve hub was pressed against the anus, creating a seal. A straight 35 mm laparoscopic grasper can be seen sliding beside the optical trocar into the anus. The seal is maintained by pressure from the anal sphincter.

The patient was placed in lithotomy position and a 12 mm blunt-tip laparoscopic trocar (Endopath Excel, Ethicon, Somerville, NJ, USA) was inserted into the anus. The trocar sleeve was advanced fully until the adjustable sleeve hub was pressed against the anus, creating a seal (Figure 2). Two silk sutures were inserted into the gluteal skin at 3 and 9 o'clock and tied to the suture posts to secure the trocar. The resultant seal was sufficient to allow insufflation, enabling insertion of a standard 35 mm 0° laparoscope.

**Figure 3:**
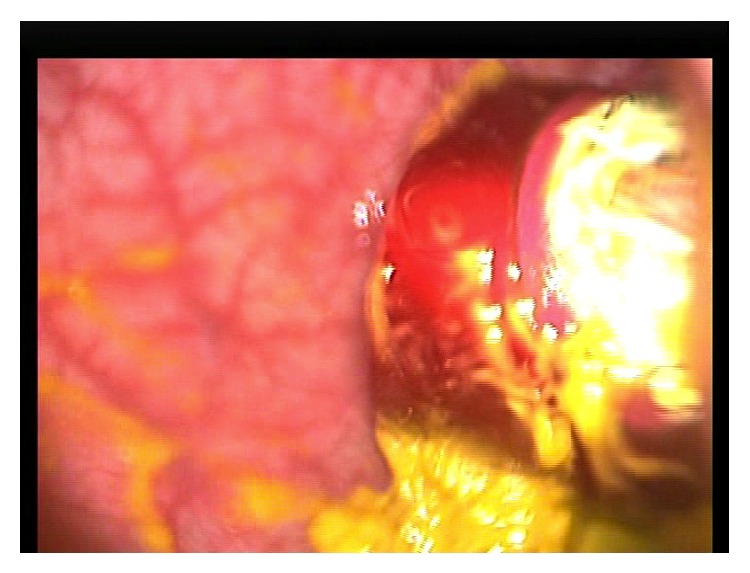
The cigar sheath is visualized in the sigmoid colon and is oriented such that the open base is firmly apposed to the sigmoid mucosa, creating a vacuum effect.

**Figure 4:**
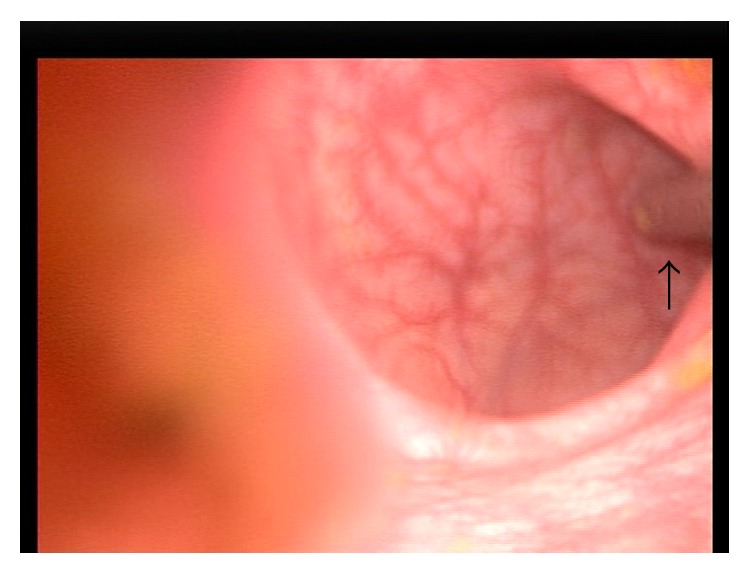
In order to retrieve the object, a straight 35 mm laparoscopic grasper was introduced into the lumen and advanced under laparoscopic vision (arrow) in order to avoid iatrogenic injury to the mucosa.

**Figure 5:**
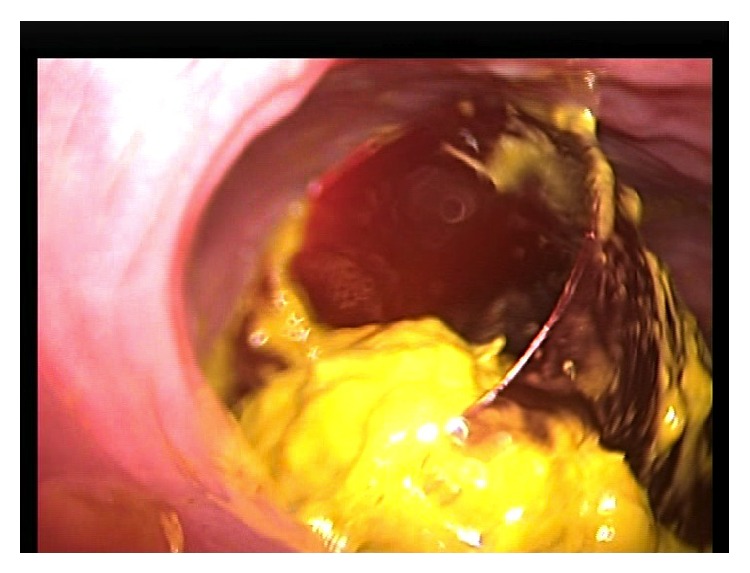
The instruments have been used to manipulate the cigar sheath and to overcome the vacuum effect. The dislodged cigar case has been rotated within the lumen so that the rim now faces the laparoscope.

**Figure 6:**
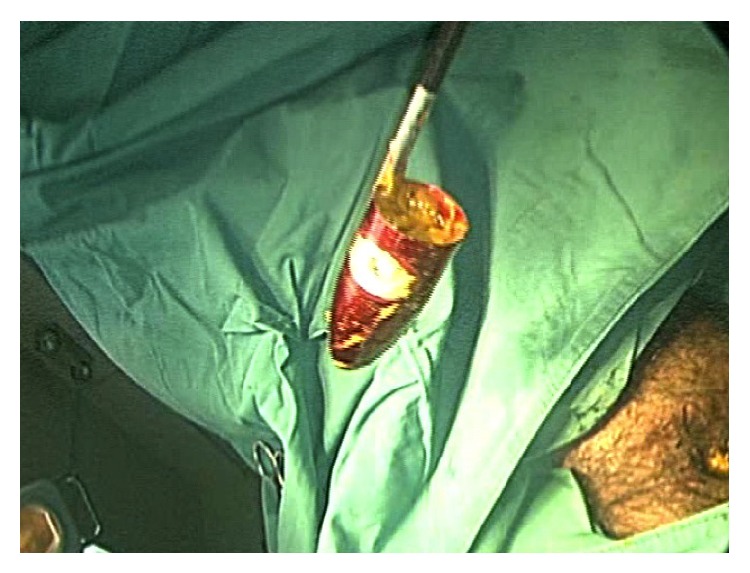
Laparoscopic graspers were used to grasp the rim of the cigar sheath and extract it under laparoscopic vision.

The cigar sheath was visualized in the sigmoid 30 cm proximal to the anus. It was oriented such that its base faced distally with the open rim firmly apposed to the sigmoid mucosa by vacuum effect (Figure 3). In an attempt to retrieve the CFB, two 35 mm straight laparoscopic graspers were slid beside the optical trocar into the anus (Figure 2). Apart from the optical trocar, no additional trocars were used. In order to prevent iatrogenic injury, we navigated the laparoscopic graspers into the sigmoid under laparoscopic vision (Figure 4). The instruments were sufficiently sturdy to allow the cigar sheath to be manipulated, overcoming the vacuum effect and dislodging it from the mucosa. This allowed the cigar case to be rotated within the colonic lumen so that the rim now faced the laparoscope (Figure 5). The laparoscopic graspers were used to grasp the rim of the cigar sheath and the CFB was extracted under vision (Figure 6).

The patient recovered uneventfully and was spared laparotomy with the concomitant morbidity.

## 3. Discussion

In modern practice, CFBs are not uncommon although the true incidence remains unsettled because of inconsistent reporting. In our case, the patient initially refused to divulge the relevant history because he was embarrassed. This was not surprising since it has been established that many patients with CFB are deceptive historians [2, 3]. As many as 20% of patients will not divulge their history of CFB insertion at presentation [4] because the practice is still considered taboo. To overcome this barrier, clinicians should approach these patients in a candid manner in order to earn their trust during history taking.

An accurate history is important to ascertain the diagnosis because any delay increases the risk of complications, such as perforation or bleeding [3]. An accurate history is also important to plan the therapeutic approach. In retrospect, we could have predicted failure of endoscopic retrieval. Most reports of endoscopic retrieval involve encircling a part of the CFB with endoscopic polypectomy snares [3, 5–7]. However, there would have been no conceivable point to grasp a smooth, tapered cigar sheath with polypectomy snares.

Operative retrieval by either milking the CFB toward the anus [3, 8, 9] or by extracting via colostomy [10–12] has been described and is accepted therapeutic methods. However, they are both accompanied by attendant morbidity from laparotomy or laparoscopy. A transanal approach, when feasible, does not require a breach of the peritoneal cavity and therefore avoids potential formation of intraperitoneal collections, organ space infections, anastomotic leaks, and damage to surrounding viscera.

Transanal extraction has been documented to be successful in 60–75% of cases [3, 12–14]. In this case, transanal retrieval was not feasible because the CFB had ascended into the sigmoid colon. We were not surprised since there is free communication between the rectal and colonic lumina, with nothing to prevent retrograde migration of luminal contents. An alternative method that has been described to assist transanal extraction is to use a Foley catheter [3]. The catheter is advanced proximal to the CFB and then inflated. The inflated catheter is slowly withdrawn, pulling the CFB with it until it comes into grasp of the examining fingers. This might have yielded success in our case, but it was not considered at the time.

We successfully utilized a modified TAMIS technique in this case. TAMIS was initially conceptualized to facilitate complete excision of upper rectal neoplasms that could not be extracted transanally [15]. This approach could theoretically spare patients the morbidity associated with anterior resections, including sexual dysfunction, urinary retention, and loss of the anal sphincter complex [15, 16]. In addition, it allows dissection under magnification leading to more accurate dissection and better hemostasis [15, 17].

There are several limitations, however, to widespread use of TAMIS including the requirement for specialized instruments, TAMIS access platforms, and a steep learning curve [15]. Some have also raised concern about post-TAMIS faecal incontinence with use of the TAMIS 40 mm rigid proctoscope [16]. Many of these concerns were overcome by using the SILS port (Covidien, Mansfield, MA, USA) to perform TAMIS [17, 18]. However, this is a limitation in a resource poor environment such as ours. In our setting, each SILS port costs US $470.21 from local distributors [19].

We believe that this modified TAMIS technique was better suited to a resource poor environment because there is no need for specialized instruments, TAMIS proctoscopes or SILS ports. The cost is contained because only standard laparoscopic instrumentation is used. It also provides a unique opportunity to repair any injuries using intraluminal laparoscopic suturing, requiring no additional skill sets compared to conventional laparoscopy. In this modified TAMIS technique, we used a single visual trocar and the working instruments were slid beside the trocar. This idea was inspired by the previously described direct transfascial puncture technique for single incision laparoscopy where instruments were slid along the working trocar without additional working ports [19]. However, sliding the instruments between the anal sphincter and the trocar did result in disruption of the air seal. Three manoeuvers were used to overcome this: we advanced the trocar fully into the rectum until the adjustable hub pressed against the anal sphincter, sutures were placed around the suture posts to maintain the seal, and the surgical assistant gently pressed the adjustable hub onto the anal sphincter during the procedure.

## 4. Conclusions

Patients with retained CFB are increasingly presenting to emergency rooms. Examination under anaesthesia and attempts at transanal extraction are important first line therapeutic steps. However, when these fail, a modified TAMIS technique can be considered using standard trocars and instruments. This is safe and cost effective and requires no specialized equipment or expertise to perform.
